# Adsorption Separation of Analgesic Pharmaceuticals from Ultrapure and Waste Water: Batch Studies Using a Polymeric Resin and an Activated Carbon

**DOI:** 10.3390/polym10090958

**Published:** 2018-08-29

**Authors:** Ricardo N. Coimbra, Carla Escapa, Marta Otero

**Affiliations:** 1 Department of Applied Chemistry and Physics, Institute of Environment, Natural Resources and Biodiversity (IMARENABIO), Universidad de León, 24001 León, Spain; ricardo.decoimbra@unileon.es (R.N.C.); carla.escapa@unileon.es (C.E.); 2 Centre for Environmental and Marine Studies (CESAM), Department of Environment and Planning, University of Aveiro, 3800 Aveiro, Portugal

**Keywords:** emerging contaminants, medicines, paracetamol, sewage, waste water treatment

## Abstract

The performance of a polymeric resin (Sepabeads SP207, from Resindion, Binasco, Italy) was compared with that of an activated carbon (GPP20, from Chemviron Carbon, Feluy, Belgium) in the adsorption of acetaminophen and ibuprofen from either ultrapure or waste water. Kinetic and equilibrium adsorption experiments were carried out under batch operation conditions, and fittings of the obtained results to different models were determined. The kinetic experimental results fitted the pseudo-first and -second order equations, and the corresponding kinetic rates evidenced that the pharmaceuticals adsorption was faster onto GPP20 than onto Sepabeads SP207, but was mostly unaffected by the aqueous matrix. The equilibrium results fitted the Langmuir-Freundlich isotherm model. The corresponding maximum adsorption capacity (*Q*_m_, mg^−1^) was larger onto GPP20 (202 mg g^−1^ ≤ *Q*_m_ ≤ 273 mg g^−1^) than onto the polymeric resin (7 mg g^−1^ ≤ *Q*_m_ ≤ 18 mg g^−1^). With respect to the parameter *K*_LF_ (mg g^−1^ (mg L^−1^)^−1/n^), which points to the adsorbent-adsorbate affinity, greater values were determined for the pharmaceuticals adsorption onto GPP20 than onto Sepabeads SP207. For both adsorbents and pharmaceuticals, neither *Q*_m_ or *K*_LF_ were affected by the aqueous matrix.

## 1. Introduction

Emerging contaminants (ECs) may be defined as compounds that are not currently covered by existing water regulations but are thought to be a threat to environmental ecosystems and human health [1]. Amongst them, pharmaceuticals constitute a group of large concern, since they were designed to trigger a physiological response. Therefore, the presence of pharmaceuticals in the aquatic environment, even at relative low concentrations, may affect non-target individuals and species.

ECs may enter the environment by different routes, depending on their usage pattern and on their application mode. In the specific case of pharmaceuticals that come from human consumption and/or excretion, municipal waste water treatment plants (WWTPs) are main sources in the aquatic environment [1]. These plants are mostly inefficient in the removal of these pollutants since the primary and secondary treatments usually applied were not designed for this purpose, but for the removal of regulated parameters [2]. However, since legislation on the discharge of pharmaceuticals is expected to come out in the near future [3], it is necessary to find efficient treatments.

Removal of pharmaceuticals at WWTPs before discharge would be possible by the inclusion of an appropriate tertiary treatment [4]. Among the different options, adsorptive treatments are convenient due to their low energy consumption and simple operation [5]. Furthermore, the incorporation of adsorptive treatments into current WWTPs is quite feasible [6]. Recently, Akhtar et al. [7] reviewed the literature on the adsorption and adsorbents used for the removal of pharmaceuticals from water. Although activated carbon is the most frequently used adsorbent for this purpose [8], polymeric materials have been also employed in such applications [9,10,11,12,13,14]. Polymeric adsorbents have several advantages over conventional activated carbons: their simpler processing; considerably easier regeneration; the possibility to shape them into the most suitable form (e.g., sheets, beads, membranes); the potential of controlling the pore structure and internal surface area by varying the polymerization conditions [15]; and the possible modification of their physical and chemical properties [16]. In any case, and whatever the adsorbent, a main advantage of adsorption processes for the removal of pharmaceuticals from water is that they do not entail the generation of transformation products [17,18,19], whose concentration and/or toxicity may be larger than for the parent compound.

Anti-inflamatory drugs are frequently detected in natural waters due to their widespread consumption [20]. In fact, acetaminophen and ibuprofen are among the most widely used drugs for fever and headaches, since they may be acquired without prescription. Due to their over-the-counter availability, they are mostly ubiquitous in municipal waste water, with high detection frequencies and relatively high concentrations compared with other drugs [21]. Indeed, in a recent work by Bellver-Domingo et al. [22], the shadow price for the WWTPs discharge of several ECs was evaluated in three ecosystems, namely wetlands, river, and sea. The aim was to weigh the environmental damage caused by ECs, which is essential information to frame the subsequent decision-making process on the management measures that should be implemented. These authors [22] found that, for the three ecosystems, acetaminophen, followed by ibuprofen, had the largest shadow prices among the studied ECs, which points to the removal of these pharmaceuticals from WWTPs effluents as a priority.

Several authors have reported the utilization of different types of adsorbents to remove acetaminophen [23,24,25] and ibuprofen [26,27,28] from water. However, just Suntisukaseam et al. [29] studied the utilization of three polymeric resins (Amberlite XAD2, XAD7, and XAD761) for the adsorption of acetaminophen from ultrapure water. Still, to our best knowledge, the utilization of polymeric resins for the adsorptive separation of acetaminophen and ibuprofen from waste water has never been assessed. In fact, the scarceness of studies on the adsorptive removal of pharmaceuticals from such a matrix (waste water) versus a majority of published results from experiments carried out in deionized or ultrapure water was recently highlighted [30].

In this context, the aim of the present work was to compare the performance of a polymeric resin and an activated carbon in the removal of two pain relievers, viz. acetaminophen and ibuprofen, both with large global consumption and occurrence in waste water. In view of the practical application of these adsorbents in the tertiary treatment of waste water, the adsorption of the considered pharmaceuticals was studied both from ultrapure water and from the secondary effluent of a municipal WWTP.

## 2. Materials and Methods

### 2.1. Adsorbent Materials

The activated carbon used in this study was pulverised GPP20, which was kindly provided by Chemviron Carbon (Feluy, Belgium). The utilization of GPP20 was recommended by Chemviron Carbon for the adsorption of pharmaceuticals, since it is an activated carbon suitable for waste water treatment applications, namely, for the removal of aromatic compounds. The polymeric resin Sepabeads SP207 (Mitsubishi Chemical Corp., Tokyo, Japan) was gently offered by Resindion (Binasco, Italy). Resindion pointed to Sepabeads SP207 as an appropriate solution for the adsorption of aromatic compounds, including pharmaceuticals. On the other hand, according to the manufacturer (Mitsubishi Chemical Corp., Tokyo, Japan), and due to its high specific gravity, this resin is appropriate not only for fixed-bed, but also for batch adsorption operation. The adsorbent materials here used were selected for this work because they have already been used for the removal of pharmaceuticals and/or other organic pollutants from water (including waste water) [9,10,31,32]. Table 1 shows the physical characteristics of these adsorbents.

### 2.2. Chemicals and Analytic Methods

Acetaminophen (≥99%) was purchased from Sigma–Aldrich (Steinheim, Germany), while ibuprofen sodium (≥98%) was purchased from Fluka (Buchs, Switzerland). The main properties of these pharmaceuticals are depicted in Table 2.

The target pharmaceuticals were analysed by High Performance Liquid Chromatography (HPLC), using a Waters HPLC 600 equipped with a 2487 Dual λ Absorbance Detector, a phenomenex C18 column (5 μm, 110 Å, 250 × 4.6 mm), a Rheodyne injector and a 50 μL loop. The wavelengths of detection were 246 and 220 for acetaminophen and ibuprofen, respectively. The mobile phase consisted of a mixture of acetonitrile:water (30:70, *v*/*v*) for the analysis of acetaminophen and a mixture of methanol:water:orthophosphoric acid (75:25:0.3) for the analysis of ibuprofen. For the preparation of the mobile phase mixtures, HPLC quality acetonitrile (CH_3_CN) and methanol (CH_3_OH) were acquired from Sigma-Aldrich (Madrid, Spain), orthophosphoric acid (H_3_PO_4_) was purchased from Panreac (Barcelona, Spain), and ultrapure water obtained by a Milli-Q purification system from Merck Millipore (Darmstadt, Germany). Before use, each mixture was passed through a Millipore 0.45 µm pore size filter and degasified in an ultrasound bath for 30 min. For the chromatographic determination of concentration, four replicated injections were carried out under a mobile phase flow rate of 1 mL min^−1^.

### 2.3. Aqueous Matrices for Adsorption Experiments

In this work, adsorption experiments were first carried out in ultrapure water (conductivity = 55 μS·cm^−1^, resistivity = 18.18 MΩ.cm and pH = 6.9 at 25 °C), which was sourced by a Milli-Q purification system. Then, aiming to assess the possible utilisation of the considered adsorbents in tertiary waste water treatment, experiments on the adsorption of pharmaceuticals from real waste water were carried out. For this purpose, the secondary effluent from the municipal waste water treatment plant (WWTP) of León (Spain) was collected. This WWTP consists of a primary stage, followed by a secondary stage treatment. The primary stage comprises screening, sand removal, fat removal, and primary clarification treatments. Then, the secondary stage involves a plug-flow activated sludge with nitrification/denitrification followed by a secondary clarification. After the secondary clarification, the effluent is directly discharged at the Bernesga river, which has a length of 77 km, and is a tributary of the Esla river. The WWTP was designed to treat the waste water of 330,000 equivalent inhabitants, and has an inflow of 123,000 m^3^ day^−1^, with a hydraulic retention time (HRT) of about 6 h.

Waste water quality parameters, namely pH, conductivity, total suspended solids (TSS), biological oxygen demand at five days (BOD_5_), chemical oxygen demand (DQO), NTK, N–NH_4_, N–NO_3_, N–NO_2_, total P–PO_4_, were determined according to Standard Methods [33]. The determined parameters are shown in Table 3, together with European regulations on the discharge of this sort of effluents, which, as may be seen, are fulfilled.

### 2.4. Adsorption Experiments

Adsorption experiments were done under batch operation conditions. For each pharmaceutical, adsorption kinetic experiments were first carried out in order to determine the time necessary to attain equilibria (*t*_eq_). Next, equilibrium experiments were done to determine the corresponding adsorption isotherm. All experiments were carried out by shaking (250 rpm) a known mass of adsorbent (Sepabeads SP207 or GPP20) together with 100 mL of ultrapure or waste water in 250 mL Erlenmeyer flasks. Initial concentration of each target pharmaceutical in waste water was 100 ± 1 mg L^−1^. All experiments were done in triplicate and at a constant temperature of 25 ± 2 °C, provided by a thermostatically regulated shaker. Triplicate controls, which consisted of solutions of the corresponding pharmaceutical (100 ± 1 mg L^−1^) in the absence of adsorbent, were run in parallel with adsorption experiments.

In the kinetic experiments, Erlenmeyer flasks were progressively withdrawn from the shaker after pre-set time intervals. Then, from each flask, three aliquots were taken, filtered, and chromatographically analyzed to determine the concentration of the target pharmaceutical. For each of the pharmaceuticals here considered (acetaminophen and ibuprofen), the adsorbed concentration onto each adsorbent at any time, *q*_t_ (mg g^−1^), was calculated by the following mass balance relationship (Equation (1)):

(1) $$q_{t}=\left(C_{0}-C_{t}\right)\frac{V}{W}$$

where *C*_t_ (mg L^−1^) is the experimental liquid-phase concentration of pharmaceutical at a time t, *C*_0_ (mg L^−1^) is the average concentration of pharmaceutical in the corresponding control, *V* is the volume of the solution (L), and *W* is the mass (g) of adsorbent.

For equilibrium experiments, Erlenmeyer flasks containing the target pharmaceutical solution together with the corresponding adsorbent were under shaking during the *t*_eq_. Then, from each flask, three aliquots were withdrawn, filtered and chromatographically analyzed to determine the pharmaceutical equilibrium concentration (*C*_e_, mg L^−1^) in the liquid phase. Then, the equilibrium adsorbed concentration of pharmaceutical onto the corresponding adsorbent, *q*_e_ (mg g^−1^), was calculated by the mass balance relationship displayed in Equation (2):

(2) $$q_{e}=\left(C_{0}-C_{e}\right)\frac{V}{W}$$

where *C*_e_ (mg L^−1^) is the experimental equilibrium liquid-phase concentration of pharmaceutical.

### 2.5. Modeling of Adsorption Results

Fittings of the obtained kinetic results to the pseudo first-order [34] and the pseudo second-order [35] equations, which are next shown (Equations (3) and (4), respectively), were determined.

(3) $$q_{t}=q_{e}(1-e^{-k_{1}t})$$

(4) $$q_{t}=\frac{q_{e}^{2}k_{2}t}{1+q_{e}k_{2}t}$$

where *k*_1_ (min^−1^) and *k*_2_ (mg g^−1^ min) are the pseudo-first and the pseudo-second order rate constants, respectively.

Fittings of the obtained adsorption equilibrium results to three different isotherm models were determined, namely the Freundlich isotherm [36], the Langmuir isotherm [37] and the Sips isotherm [38], which is commonly designated as the Langmuir-Freundlich isotherm. These isotherms are next depicted by Equations (5), (6) and (7), respectively.

(5) $$q_{e}=K_{F}C_{e}^{1/n}$$

(6) $$q_{e}=\frac{Q_{m}K_{L}C_{e}}{1+K_{L}C_{e}}$$

(7) qe=QmKLFCe1n1+KLFCe1n

where *K*_F_ is the Freundlich adsorption constant (mg g^−1^ (mg L^−1 −1/n^); *n* the degree of non-linearity; *Q_m_* the maximum adsorption capacity (mg g^−1^); *K*_L_ (L mg^−1^) and *K*_LF_ (mg g^−1^ (mg L^−1^)^−1/n^) are the Langmuir and Langmuir-Freundlich affinity coefficients, respectively.

## 3. Results and Discussion

**Figure 1:**
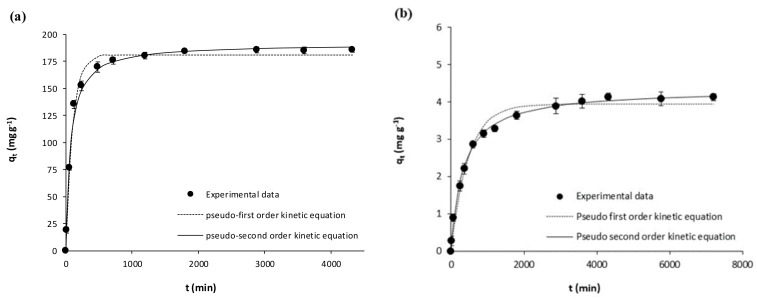
Kinetic results on the adsorptive removal of acetaminophen from ultrapure water by adsorption onto (**a**) the activated carbon GPP20 and (**b**) the polymeric resin Sepabeads SP207. Experimental results are shown together with fittings to the pseudo-first and pseudo second order kinetic equations. Error bars stand for standard deviation of three experimental replications. Note: for a better visualization of fittings, the scale of axis Y has been adjusted to results.

**Figure 2:**
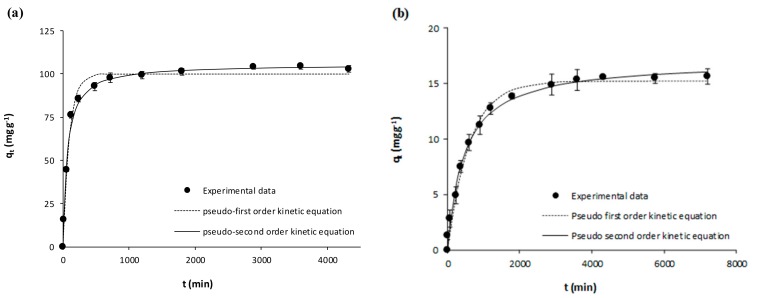
Kinetic results on the adsorptive removal of ibuprofen from ultrapure water by adsorption onto (**a**) the activated carbon GPP20 and (**b**) the polymeric resin Sepabeads SP207. Experimental results are shown together with fittings to the pseudo-first and pseudo second order kinetic equations. Error bars stand for standard deviation of three experimental replications. Note: for a better visualization of fittings, the scale of axis Y has been adjusted to results.

**Figure 3:**
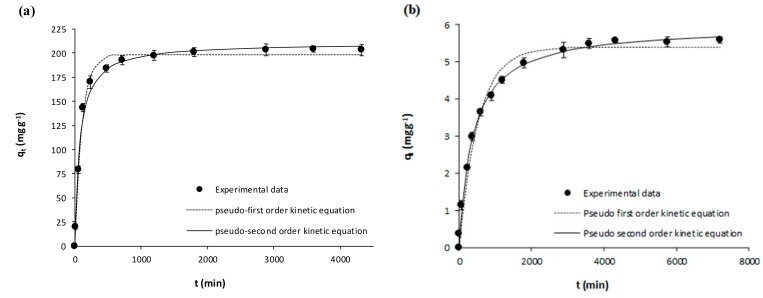
Kinetic results on the adsorptive removal of acetaminophen from waste water by adsorption onto (**a**) the activated carbon GPP20 and (**b**) the polymeric resin Sepabeads SP207. Experimental results are shown together with fittings to the pseudo-first and pseudo second order kinetic equations. Error bars stand for standard deviation of three experimental replications. Note: for a better visualization of fittings, the scale of axis Y has been adjusted to results.

**Figure 4:**
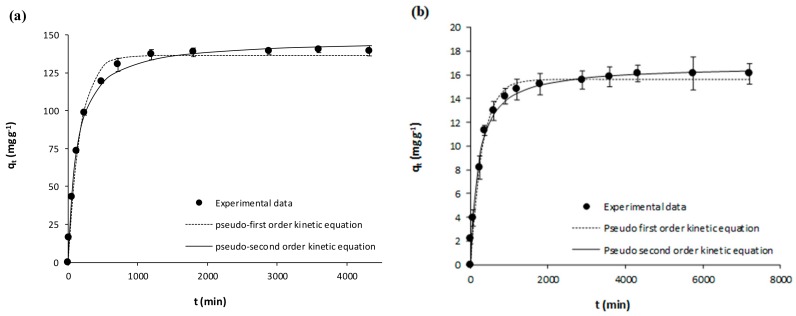
Kinetic results on the adsorptive removal of ibuprofen from waste water by adsorption onto (**a**) the activated carbon GPP20 and (**b**) the polymeric resin Sepabeads SP207. Experimental results are shown together with fittings to the pseudo-first and pseudo second order kinetic equations. Error bars stand for standard deviation of three experimental replications. Note: for a better visualization of fittings, the scale of axis Y has been adjusted to results.

The kinetic experimental results on the adsorption of acetaminophen and ibuprofen from ultrapure water onto the activated carbon (GPP20) and onto the polymeric resin (Sepabeads SP207) are represented in Figure 1 and Figure 2. Meanwhile, Figure 3 and Figure 4 represent the corresponding experimental kinetic results from waste water. In these figures, fittings to the kinetic models here considered (Equations (3) and (4)) are shown together with experimental results whereas the corresponding fitted parameters are depicted in Table 4. In Figure 1, Figure 2, Figure 3 and Figure 4, it is evidenced that *q*_t_ increases with *t* until reaching the equilibrium, and then *q*_t_ remains stable throughout time. As it may be seen in these figures, although experimental results were slightly better described by the pseudo-second equation, both the here used kinetic models may be considered appropriate, which is confirmed by the *R*^2^ (*R*^2^ ≥ 0.98). As for the corresponding fitted kinetic constants in Table 4, the adsorption of the here studied pharmaceuticals was faster onto the activated carbon than onto the polymeric resin, which must be mainly related to the larger particle size of the latter (Table 1). On the other hand, the adsorption velocity was mostly unaffected by the matrix, with similar kinetic constants for the adsorption from ultrapure and waste water. Then, for the subsequent equilibrium experiments, the *t*_eq_ values for the adsorption onto GPP20 and onto Sepabeads SP207 were 48 and 72 h, respectively.

**Figure 5:**
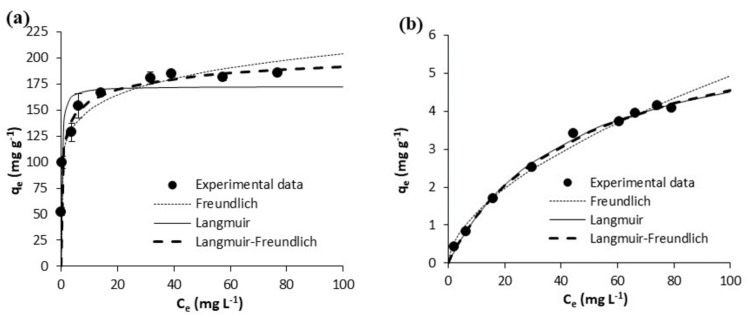
Equilibrium results on the adsorptive removal of acetaminophen from ultrapure water by adsorption onto (**a**) the activated carbon GPP20 and (**b**) the polymeric resin Sepabeads SP207. Experimental results are shown together with fittings to the Freundlich, to the Langmuir and to the Langmuir-Freundlich isotherm models. Error bars stand for the standard deviation of three experimental replications. Note: for a better visualization of fittings, the scale of axis Y has been adjusted to results.

**Figure 6:**
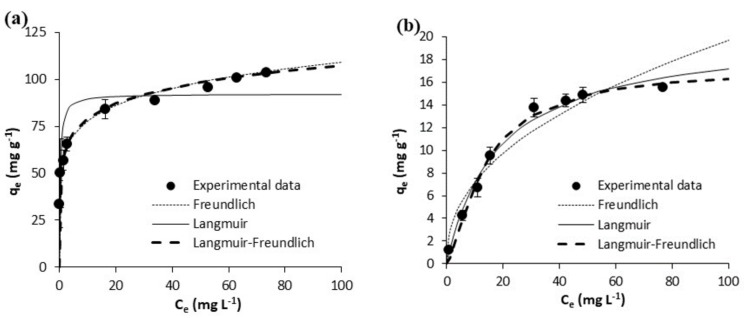
Equilibrium results on the adsorptive removal of ibuprofen from ultrapure water by adsorption onto (**a**) the activated carbon GPP20 and (**b**) the polymeric resin Sepabeads SP207. Experimental results are shown together with fittings to the Freundlich, to the Langmuir and to the Langmuir-Freundlich isotherm models. Error bars stand for the standard deviation of three experimental replications. Note: for a better visualization of fittings, the scale of axis Y has been adjusted to results.

**Figure 7:**
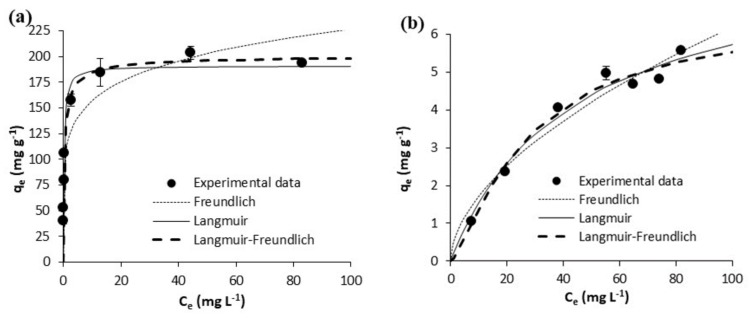
Equilibrium results on the adsorptive removal of acetaminophen from waste water by adsorption onto (**a**) the activated carbon GPP20 and (**b**) the polymeric resin Sepabeads SP207. Experimental results are shown together with fittings to the Freundlich, to the Langmuir and to the Langmuir-Freundlich isotherm models. Error bars stand for the standard deviation of three experimental replications. Note: for a better visualization of fittings, the scale of axis Y has been adjusted to results.

**Figure 8:**
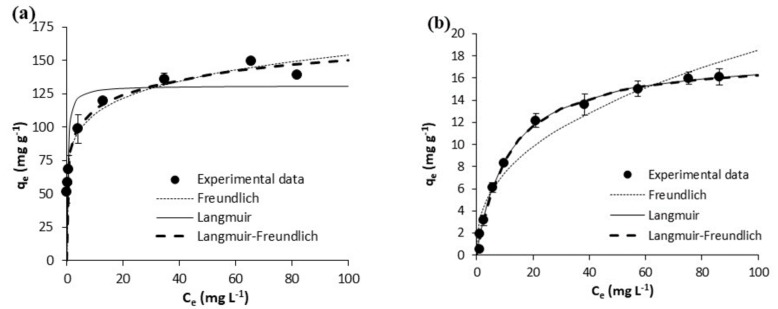
Equilibrium results on the adsorptive removal of ibuprofen from waste water by adsorption onto (**a**) the activated carbon GPP20 and (**b**) the polymeric resin Sepabeads SP207. Experimental results are shown together with fittings to the Freundlich, to the Langmuir and to the Langmuir-Freundlich isotherm models. Error bars stand for the standard deviation of three experimental replications. Note: for a better visualization of fittings, the scale of axis Y has been adjusted to results.

The equilibrium experimental results on the adsorption of acetaminophen and ibuprofen from ultrapure water are shown in Figure 5 and Figure 6, respectively. Those on the adsorption of acetaminophen and ibuprofen from waste water are respectively depicted in Figure 7 and Figure 8. Equilibrium data are represented in these figures, together with fittings to the considered isotherm models (Equations (5)–(7)); the corresponding fitted parameters are depicted in Table 5.

As may be seen in Figure 5, from ultrapure water, the adsorbed concentrations of acetaminophen at the equilibrium were larger onto GPP20 than onto Sepabeads SP207, which must be largely due to the greater specific surface area of the first (Table 1). In agreement, larger adsorption concentrations at the equilibrium were also determined for ibuprofen onto GPP20 than onto the polymeric resin.

When comparing the adsorption of acetaminophen and ibuprofen, larger *q*_e_ were determined for acetaminophen than for ibuprofen onto the activated carbon GPP20, while the opposite was observed in the case of Sepabeads SP207. Since pH effects strongly affect the adsorption process in general and the adsorption capacity in particular, this difference must be related to the different pKa values of the pharmaceuticals (Table 2). According to these values, acetaminophen is neutral at the pH of ultrapure water (pH = 6.9), while ibuprofen is negatively charged. Therefore, in the case of acetaminophen, the main factor conditioning its binding is the occurrence of non-electrostatic interactions such as hydrogen-bonds and Van deer Waals-forces. Differently, the adsorption of ibuprofen is affected by electrostatic interactions. In fact, the point of zero charge (PZC) of GPP20 and Sepabeads SP207 were determined to be 7.4 and 8.6, respectively. Hence, while the surface of GPP20 at the working pH is mostly uncharged, that of the polymeric resin is positively charged, which explains its higher affinity and capacity for ibuprofen (negative charged form) than for acetaminophen (neutral form).

In waste waster (Figure 7 and Figure 8), the adsorption equilibrium isotherms remained mostly the same as those in ultrapure water, with the above observations being also applicable. Yet, at the pH of waste water (7.8), acetaminophen and ibuprofen are neutral and negatively charged, respectively. On the other hand, the surface of the GPP20 is mostly neutral, and that of Sepabeads SP207, positively charged. With respect to the isotherm modeling, as may be seen in Figure 5, Figure 6, Figure 7 and Figure 8, the best fittings were to the Langmuir-Freundlich model. This is confirmed by *R*^2^ and *S*_xy_ values in Table 5, which make evident the differences between fittings to the different isotherm models.

As may be seen in Table 5, according to fittings to the Langmuir-Freunlich isotherm, the maximum adsorption capacity (*Q*_m_) values obtained for acetaminophen onto GPP20 were two orders of magnitude larger than onto Sepabeads SP207. Then, for each adsorbent, equivalent *Q*_m_ were obtained for the adsorption of acetaminophen from ultrapure and from waste water. In the case of ibuprofen, the Langmuir-Freundlich fitted *Q*_m_ values were one order of magnitude larger onto GPP20 than onto Sepabeads SP207. Also, for each adsorbent, similar *Q*_m_ were determined for the adsorption of ibuprofen from ultrapure and from waste water. These results are in agreement with those by Suntisukaseam et al. [29], who also found that the adsorption capacity of activated carbon was larger than that of several polymeric resins in the removal of pharmaceuticals (acetaminophen and nalidixic acid) from water.

According to the fitted Langmuir-Freudnlich isotherms, the average *Q*_m_ determined for the adsorption of ibuprofen were larger than for the adsorption of acetaminophen for both of the presently-used adsorbents This is apparently in disagreement with the larger *q*_e_ that had been experimentally determined for the adsorption of acetaminophen onto GPP20, as compared with ibuprofen. However, it must be highlighted that large standard deviations were observed for the *Q*_m_ values determined for ibuprofen. In fact, although all the isotherms fitted the Langmuir-Freunlich isotherm, those corresponding to the adsorption of acetaminophen onto GPP20 reached a well-defined plateau, which was not the case of ibuprofen. This further supports that, in contrast to ibuprofen, the adsorption of acetaminophen is governed by non-electrostatic interactions.

Regarding the *K*_LF_, a parameter that is frequently related to the affinity of the adsorbent for the adsorbate, larger values were determined for GPP20 than for Sepabeads SP207. In the case of GPP20, a larger affinity for acetaminophen than for ibuprofen may be inferred on the basis of *K*_LF_ values, which agrees with the neutral charge of GPP20 and acetaminophen at the working pH (both in ultrapure and waste water). Meanwhile, Sepabeads, which is positively charged at the working pH, showed larger affinity for ibuprofen (negative form) than for acetaminophen (neutral).

It is to be noted that, in this work, neither the *q*_e_ nor the *Q*_m_ values decreased in waste water as compared to ultrapure water, which points to the applicability of both the activated carbon and the polymeric resin for an adsorptive tertiary treatment of waste water.

In the literature, there are published works (ex: [39,40]) that report the reduction of the pharmaceuticals adsorption uptake by carbonaceous adsorbents in waste water as compared with ultrapure or distilled water, pointing to the competitive effects of dissolved organic matter (DOM). In contrast, there are studies (ex: [41]) that highlight an increased adsorption capacity of organic pollutants in waste water, and relate this increase to the action of microorganisms in waste water, the adsorbent acting as a bearer surface that favors their degrading. In this sense, Combarros et al. [42] proved that the formation of a bacterial biofilm on the surface of a commercial activated carbon meant the increase of the adsorption of salicylic acid from water. Therefore, in this work, it may be hypothesized the occurrence of a synergism between microorganisms degradation and adsorption for the removal of pharmaceuticals. Thus, the action of microorganisms may have compensated for the competitive effect by DOM so the pharmaceuticals adsorption capacity remained the same in waste water than in ultrapure water.

## 4. Conclusions

Both the polymeric resin Sepabeads SP207 and the activated carbon GPP20 were able to adsorb acetaminophen and ibuprofen from ultrapure and waste water. Adsorption kinetics were faster onto GPP20 than onto Sepabeads SP207, but were mostly unaffected by the aqueous matrix. Experimental results fitted both the pseudo-first and the pseudo-second kinetic models, the latter giving slightly higher correlation coefficients. Equilibrium results onto both the considered adsorbents and for both pharmaceuticals fitted the Langmuir-Freundlich isotherm model, either from ultrapure or from wastewater. The activated carbon displayed larger adsorption capacity values at the equilibrium (202 mg g^−1^ ≤ *Q*_m_ ≤ 273 mg g^−1^) than the polymeric resin (7 mg g^−1^ ≤ *Q*_m_ ≤ 18 mg g^−1^), which was especially remarkable in the case of acetaminophen. Still, the adsorption capacity of these pharmaceuticals onto the employed adsorbents did not decrease in waste water with respect to ultrapure water. This is a very important finding, which points to the practical application of GPP20 and Sepabeads 207 in the tertiary treatment of waste water.

## Figures and Tables

**Table 1:** Physical properties of adsorbents used for diclofenac acid adsorption.

Adsorbent	GPP20	Sepabeads SP207
Matrix	Coal based steam activated carbon	Styrene-divinylbenzene copolymer
Colour	Black carbon	Yellowish opaque beads
Specific surface area (m^2^ g^−1^)	1000	650
Mean particle diameter (mm)	0.04	0.4

**Table 2:** Physico-chemical properties of the pharmaceuticals used in this study (Source: Chemspider and Chemicalize).

Pharmaceutical (Formula)	Structure	Mw (g mol^−1^)	Sw ^a^ (mg L^−1^)	pKa	Log K_ow_	PSA (A^2^)	HBAC
**Ibuprofen Sodium (C_3_H_17_NaO_2_)**	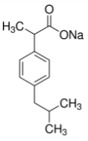	228.26	100,000	4.91	3.8	40.1	2
**Acetaminophen (C_8_H_9_NO_2_)**	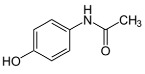	151.17	14,000	9.48	0.46	49.3	2

PSA = Polar Surface Area, HBAC = Hydrogen Bound Acceptor Count, ^a^ Sw = water solubility (25 °C).

**Table 3:** Main properties of the urban WWTP effluent used in this work.

pH	Conductivity	TSS	BOD_5_	COD	NTK	N–NH_4_	N–NO_3_	N–NO_2_	Total P–PO_4_
	(µS cm^−1^)	(mg L^−1^)	(mg L^−1^)	(mg L^−1^)	(mg L^−1^)	(mg L^−1^)	(mg L^−1^)	(mg L^−1^)	(mg L^−1^)
7.8 ± 0.2	612 ± 3	22 ± 1	21 ± 2	47 ± 3	17 ± 2	13.10 ± 0.42	1.73 ± 0.18	0.48 ± 0.09	1.75 ± 0.13
		35 *	25 *	125 *					

* Limits for discharge according to the Urban Waste Water Directive (Council Directive 91/271/EEC of 21 May 1991 concerning urban waste water treatment as amended by Commission Directive 98/15/EC and Regulations 1882/2003/EC and 1137/2008/EC).

**Table 4:** Kinetic parameters obtained from the fittings of experimental results on the adsorption of acetaminophen and ibuprofen from ultrapure water (UPW) and from waste water (WW) to the pseudo-first and to the pseudo-second order equations.

Parameters		Acetaminophen				Ibuprofen			
		GPP20		SP207		GPP20		SP207	
		UPW	WW	UPW	WW	UPW	WW	UPW	WW
**Pseudo-first Order**	***k*_1_ (min^−1^)**	0.0098 ± 0.0007	0.0093 ± 0.0006	0.0021 ± 0.0002	0.0019 ± 0.0002	0.0105 ± 0.0009	0.0058 ± 0.0004	0.0016 ± 0.00001	0.0033 ± 0.0003
	***q*_e_ (mg g^−1^)**	180.60 ± 2.48	198.20 ± 2.56	3.95 ± 0.09	5.39 ± 0.11	99.95 ± 1.63	136.60 ± 2.04	15.24 ±0.27	15.63 ± 0.23
	***R*^2^**	0.9914	0.9925	0.9800	0.9832	0.9872	0.9914	0.9889	0.9849
	***S_xy_***	6.59	6.76	0.22	0.27	4.35	5.04	0.63	0.73
**Pseudo-second Order**	***k*_2_ (g mg^−1^ min^−1^)**	0.00008 ± 0.00001	0.00006 ± 0.00001	0.00069 ± 0.00005	0.00045 ± 0.00003	0.00015 ± 0.00001	0.00006 ± 0.000004	0.00013 ± 0.00001	0.00031 ± 0.00003
	***q*_e_ (mg g^−1^)**	191.20 ± 2.99	210.30 ± 3.40	4.34 ± 0.05	5.96 ± 0.08	105.60 ± 1.49	146.90 ± 1.75	17.04 ± 0.32	16.77 ± 0.27
	***R*^2^**	0.9916	0.9913	0.9961	0.9958	0.9926	0.9961	0.9927	0.9911
	***S*_xy_**	6.48	7.30	0.10	0.13	3.29	3.39	0.51	0.55

**Table 5:** Isotherm parameters obtained from the fittings of the equilibrium experimental results on the adsorption of acetaminophen and ibuprofen from ultrapure water (UPW) and from waste water (WW) to the isotherm models of Freundlich, Langmuir and Langmuir-Freundlich.

	Parameters	Acetaminophen				Ibuprofen			
		GPP20		SP207		GPP20		SP207	
		UPW	WW	UPW	WW	UPW	WW	UPW	WW
**Freundlich**	***K*_F_ [mg g^−1^ (mg L^−1^)^−1/n^]**	110.10 ± 5.42	108.60 ± 10.44	0.36 ± 0.06	0.47 ± 0.16	55.30 ± 1.51	78.39 ± 2.88	2.60 ± 0.65	2.97 ± 0.54
	***N***	7.45 ± 0.81	6.27 ± 1.12	1.75 ± 0.12	1.79 ± 0.27	6.78 ± 0.36	6.80 ± 0.49	2.27 ± 0.35	2.51 ± 0.30
	***R*^2^**	0.9519	0.8761	0.9878	0.947	0.9865	0.9788	0.9342	0.9491
	***S*_y*x*_**	10.85	24.97	0.1704	0.41	3.19	6.16	1.52	1.45
**Langmuir**	***Q*_m_(mg g^−^^1^)**	172.40 ± 8.34	191.20 ± 5.81	6.43 ± 0.28	8.25 ± 1.01	92.26 ± 5.60	131.40 ± 9.66	20.33 ± 1.16	18.17 ± 0.75
	***K*_L_(L mg^-1^)**	4.181 ± 2.019	3.688 ± 0.545	0.023 ± 0.002	0.023 ± 0.006	3.273 ± 1.447	3.144 ± 1.448	0.054 ± 0.009	0.088 ± 0.006
	***R*^2^**	0.8285	0.9772	0.9964	0.9714	0.7751	0.7729	0.857	0.9967
	***S*_yx_**	20.47	10.72	0.09326	0.30	13.00	20.16	0.70	0.37
**Langmuir-Freundlich**	***Q*_m_ (mg g^−1^)**	235.00 ± 26.83	201.70 ± 7.49	6.90 ± 1.12	6.53 ± 1.26	260.10 ± 108.70	272.90 ± 133.30	17.25 ± 1.13	17.82 ± 0.68
	***K*_LF_ [mg g^−1^ (mg L^−1^)^−1/n^]**	0.954 ± 0.254	2.055 ± 0.471	0.025 ± 0.004	0.073 ± 0.010	0.273 ± 0.283	0.414 ± 0.298	0.026 ± 0.011	0.084 ± 0.040
	***N***	2.99 ± 0.52	1.39 ± 0.19	1.06 ± 0.12	0.76 ± 0.22	4.88 ± 1.55	4.23 ± 1.527	0.71 ± 0.11	0.96 ± 0.070
	***R*^2^**	0.9852	0.9890	0.9965	0.9760	0.9880	0.9844	0.9920	0.9968
	***S*_yx_**	6.49	8.15	0.10	0.31	3.24	5.80	0.58	0.39
